# Association between low-density lipoprotein cholesterol and sudden cardiac arrest in people with diabetes mellitus

**DOI:** 10.1186/s12933-023-01769-9

**Published:** 2023-02-20

**Authors:** Yun Gi Kim, Joo Hee Jeong, Kyung-Do Han, Seung-Young Roh, Kyongjin Min, Hyoung Seok Lee, Yun Young Choi, Jaemin Shim, Jong-Il Choi, Young-Hoon Kim

**Affiliations:** 1 Division of Cardiology, Department of Internal Medicine, Korea University College of Medicine and Korea University Anam Hospital, Seoul, Republic of Korea 73 Goryeodae-Ro, Seongbuk-Gu, 02841; 2 Department of Statistics and Actuarial Science, Soongsil University, Seoul, Republic of Korea; 3 Division of Cardiology, Department of Internal Medicine, Korea University College of Medicine and Korea University Guro Hospital, Seoul, Republic of Korea; 4 Division of Cardiology, Sanggye Paik Hospital, Inje University College of Medicine, Seoul, Republic of Korea

**Keywords:** Lipoproteins, LDL, Diabetes mellitus, Sudden cardiac arrest

## Abstract

**Background:**

Dyslipidemia measured as low-density lipoprotein (LDL)-cholesterol is an established risk factor of cardiovascular disease, which is more pronounced in diabetes population. Less is known about the association of LDL-cholesterol level and sudden cardiac arrest (SCA) risk in diabetes mellitus patients. This study investigated the association of LDL-cholesterol level and SCA risk in diabetes population.

**Methods:**

This study was based on Korean National Health Insurance Service database. Patients who received general examination from 2009 to 2012 and diagnosed as type 2 diabetes mellitus were analyzed. Primary outcome was defined as SCA event identified with International Classification of Disease code.

**Results:**

A total of 2,602,577 patients were included, with total follow-up duration of 17,851,797 person * year. Mean follow-up duration was 6.86 years, and 26,341 SCA cases were identified. Overall incidence of SCA was highest in the lowest LDL-cholesterol group (< 70 mg/dL) and decreased in a linear manner as LDL-cholesterol rises, till 160 mg/dL. Adjustment of covariates resulted in U-shape association, with highest risk of SCA in the highest LDL-cholesterol group (≥ 160 mg/dL) followed by lowest LDL-cholesterol group (< 70 mg/dL). In subgroup analysis, U-shape association between SCA risk and LDL-cholesterol was more pronounced in male, non-obese people, and those who did not use statins.

**Conclusions:**

In people with diabetes, the association between SCA and LDL-cholesterol level was U-shaped with highest and lowest LDL-cholesterol group having higher risk of SCA than others. Low LDL-cholesterol level can be a surrogate marker for increased risk of SCA in people with diabetes mellitus and this paradoxical association should be recognized and extended to clinical preventive measures.

**Supplementary Information:**

The online version contains supplementary material available at 10.1186/s12933-023-01769-9.

## Introduction

Sudden cardiac arrest (SCA) is defined as unexpected sudden loss of cardiac contraction that occurs without a warning sign [1, 2]. Although majority of SCA occurs in patients with structural heart disease, including coronary artery disease, heart failure and cardiomyopathy, SCA also affects individuals who are apparently healthy without or with few cardiovascular risk factors [3]. Interlinked with cardiovascular mortality, SCA is a significant public healthcare burden that leads to substantial socioeconomic cost globally. Although cardiovascular mortality has decreased in past two decades along with improved preventive measures, cardiovascular death still affects approximately 17 million annual deaths around the world, in which one-fourth presents as SCA [2, 4].

Considering the devastating sequalae of SCA and poor efficacy of secondary prevention, risk stratification of SCA and early recognition of high-risk population is crucial. However, due to sudden onset and dynamic nature, recognizing risk factors of SCA is an area of expertise that needs further identification. For decades, structural heart disease such as ischemic heart disease and reduced left ventricular ejection fraction have been the most widely accepted risk factors of SCA in clinical field, which leads to preventive therapy of implantable cardioverter defibrillator implantation [1, 2, 5]. Besides established structural heart disease, we have identified several independent risk factors of SCA from previous studies, which includes hypertension, diabetes mellitus, dyslipidemia, and metabolic syndrome [6, 7]. Dyslipidemia and diabetes are well-established, modifiable risk factors for future cardiovascular event. Co-existence of diabetes with dyslipidemia elevates the risk of cardiovascular disease, which is also known as diabetic dyslipidemia. Therefore, current guidelines recommend lower target of low-density lipoprotein (LDL)-cholesterol levels in people with diabetes mellitus, targeting LDL-cholesterol reduction to less than 100 mg/dL or even as low as 55 mg/dL, depending on individual risk stratification [8–10]. Although diabetes mellitus is also known as an independent predictor of SCA from previous studies, there have been conflicting results on association of dyslipidemia with SCA risk [11–15]. In the early decades, long term follow-up of Paris Prospective study demonstrated a positive association between serum cholesterol level and sudden death [11]. However, subsequent studies revealed no significant association, or even negative association between lipid level (total cholesterol or LDL-cholesterol) and SCA risk [12, 13]. Accordingly, the conflicting results in previous studies necessitated further clarification of the association between dyslipidemia and SCA risk. In addition, less is known about quantitative correlation of LDL-cholesterol level with SCA risk, as well as its inter-connection with presence of diabetes mellitus. Therefore, based on nationwide database of Korean National Health Insurance Service (K-NHIS), we sought to investigate the association between LDL-cholesterol and risk of SCA in diabetes population.

## Research design and methods

### Database

The K-NHIS is the single, exclusive medical insurance system managed by Korean government, that mandates subscription for Korean citizens, including virtually entire Korean population. A regular, nationwide health medical examination is provided for subscribers biennially, which includes physical examinations, self-reported questionnaires regarding sociodemographic factors, and laboratory test. Medical history is recorded as diagnostic codes of International Classification of Disease, 10th revision (ICD-10), and drug prescription history was recorded. This study was approved by Institutional Review Board of Korea University Medicine Anam Hospital and official review committee of the K-NHIS. Written informed consent was waived by the Institutional Review Board of Korea University Medicine Anam Hospital. This study conformed to the principle of 2013 Declaration of Helsinki.

### Study population

Patients with diagnostic codes for type 2 diabetes mellitus and those who underwent medical examination during 2009 to 2012 were included in this study. Patients (i) who were younger than 20 years, (ii) who had diagnostic codes for SCA prior to enrollment, and (iii) those with missing data were excluded. Patients were followed up from the day of initial medical examination to December, 2018. There were no follow-up losses except for death and emigrations.

### Definitions of variables

The primary outcome was defined as the occurrence of SCA during follow-up and both the aborted and non-aborted SCA events were included. Sudden cardiac arrest was identified with ICD-10 codes; I46.0 (cardiac arrest with successful resuscitation), I46.1 (sudden cardiac arrest), I46.9 (cardiac arrest, cause unspecified), I49.0 (ventricular fibrillation and flutter), R96.0 (instantaneous death), and R96.1 (death occurring less than 24 h from onset of symptoms). Out-of-hospital cardiac arrest declared at emergency department was defined as SCA event, and events during in-hospital admission were not counted. Performance of cardiopulmonary resuscitation at emergency department without claim of ICD-10 codes for SCA was also classified as a SCA event. Regarding the definition of SCA, patients with prior diagnosis of ischemic stroke, hemorrhagic stroke, asphyxia, suffocation, drowning, anaphylaxis, gastrointestinal bleeding, sepsis, major trauma, hit by lightning, electric shock, or burn within 6 months of diagnosis of SCA were excluded. The claims for SCA that occurred within 1 year after health screening was not counted as a main outcome due to ICD-10 coding-based detection of main outcome. For example, claim of SCA codes immediately after health screening can be actual SCA event after health screening or just a repeat claim of SCA which happened before health screening. Incidence of SCA was described as SCA events per 1000 person * year follow-up. The robustness of our coding strategy for SCA and other medical conditions was validated in prior studies [6, 7, 16–20].

Serum lipid profiles were measured as high-density lipoprotein-cholesterol (mg/dL), total cholesterol (mg/dL), and triglyceride (mg/dL) with at least eight hours of fasting. LDL-cholesterol (mg/dL) was calculated from quantitative measurements of total cholesterol, high-density lipoprotein-cholesterol, and triglyceride using the Friedewald formula. LDL-cholesterol level was classified into eight quantiles: LDL-cholesterol < 70 (group I), 70 ≤ LDL-cholesterol < 75 (group II), 75 ≤ LDL-cholesterol < 100 (group III), 100 ≤ LDL-cholesterol < 115 (group IV), 115 ≤ LDL-cholesterol < 130 (group V), 130 ≤ LDL-cholesterol < 145 (group VI), 145 ≤ LDL-cholesterol < 160 (group VII), 160 ≤ LDL-cholesterol (group VIII). The ICD-10 codes for diagnosis and definitions of variables are described in Additional file 1: Tables S1 and S2.

### Statistical analysis

The incidence of SCA was calculated as event numbers per 1000 person-years of follow-up. Categorical variables were described as number and percentage, and continuous variables were described as mean ± standard deviation or median and quartiles. Student’s t-test, Mann–Whitney U test, Chi-square test, and Fisher’s exact test was used for comparison of variables as indicated. Cox-proportional hazards model was used to calculate hazards ratios and 95% confidence intervals (CI). Variables that were statistically different according to LDL-cholesterol level were further included for adjusted Cox regression analysis. Consequently, adjustment with covariates was done with multivariate model 1 (age and sex) and model 2 (age, sex, income, body mass index [BMI], smoking status, alcohol consumption status, regular exercise, hypertension, fasting blood glucose, duration of diabetes mellitus, use of insulin, use of oral hypoglycemic agent, and use of statin). Our multivariate models were validated in our prior studies [6, 7, 16, 21, 22]. All tests were two-tailed, and statistical significance was defined as p-values ≤ 0.05. All statistical analyses were performed with SAS version 9.2 (SAS Institute, Cary, NC, USA).

## Results

### Study population

**Fig. 1:**
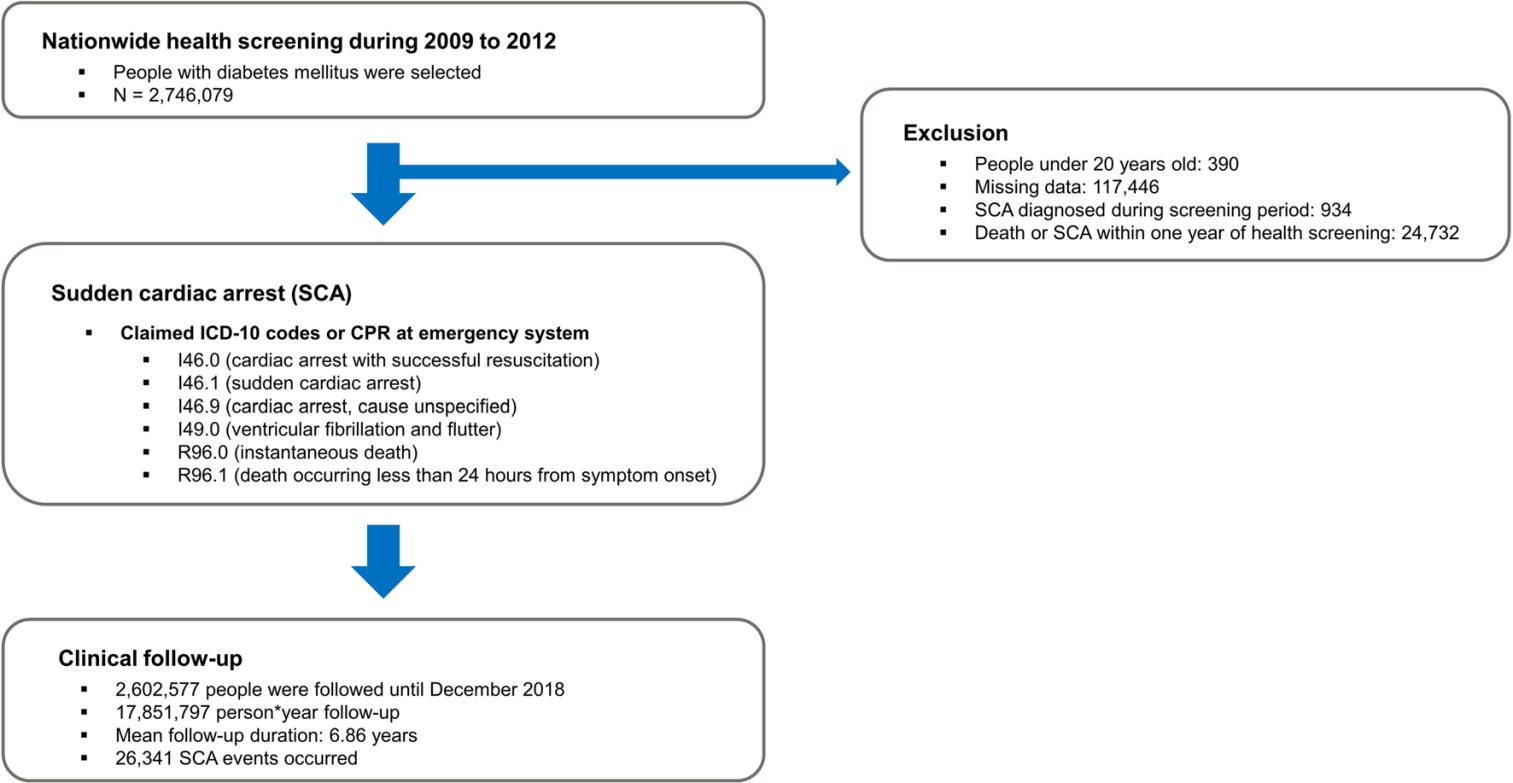
Flowsheet of study. *SCA* sudden cardiac arrest, *ICD-10* International Classification of Disease, 10th revision, *CPR* cardiopulmonary resuscitation

During 2009 to 2012, a total of 2,746,079 patients who were diagnosed with diabetes mellitus underwent nationwide medical examination (Fig. 1). Exclusion criteria were (i) Patients under 20 years (n = 390), (ii) those with missing data (n = 117,446), (iii) those with prior diagnosis of SCA during screening period (2002 to 2008; n = 934), or (iv) those who died or experienced SCA within 1 year after medical examination (n = 24,732). Patients were followed until December 2018, and total follow-up duration was 17,851,797 person * year. Mean follow-up period per patient was 6.86 years, and a total of 26,341 SCA events were identified.

Baseline characteristics regarding LDL-cholesterol level is described in Table 1. As LDL-cholesterol level decreases, there was tendency of increase of age and decrease of BMI. In addition, lower LDL-cholesterol subgroups showed higher proportion of male sex, current-smoker, heavy-drinker, and patients with regular exercise. Lower LDL-cholesterol subgroup also reflected higher proportion of pre-existing comorbidities of hypertension, and long-standing diabetes mellitus (more than 5 years), as well as prior medication of statin, multiple oral hypoglycemic agents (three or more), and insulin.

**Table 1:** Baseline characteristics according to LDL-cholesterol level

	LDL-cholesterol (mg/dL)								
	< 70	< 75	< 100	< 115	< 130	< 145	< 160	≥ 160	p-value
	343,781	305,842	389,404	415,948	380,029	297,720	203,636	266,217	
Male	235,091 (68.4%)	192,013 (62.7%)	242,681 (62.3%)	256,338 (61.6%)	227,758 (59.9%)	170,905 (57.4%)	110,355 (54.1%)	127,157 (47.7%)	
Age (years)	58.0 ± 12.1	58.2 ± 12.4	57.6 ± 12.5	57.3 ± 12.5	57.1 ± 12.4	56.9 ± 12.3	56.9 ± 12.1	56.9 ± 11.9	< 0.001
Age groups									< 0.001
< 40 years	23,448 (6.8%)	22,404 (7.3%)	30,930 (7.9%)	33,586 (8.0%)	29,972 (7.8%)	22,736 (7.6%)	15,137 (7.4%)	18,440 (6.9%)	
40–64 years	211,359 (61.4%)	182,765 (59.7%)	236,222 (60.6%)	256,037 (61.5%)	237,927 (62.6%)	189,987 (63.8%)	131,252 (64.4%)	174,977 (65.7%)	
≥ 65 years	108,974 (31.7%)	100,673 (32.9%)	122,252 (31.3%)	126,325 (30.3%)	112,130 (29.5%)	84,997 (28.5%)	57,247 (28.1%)	72,800 (27.3%)	
BMI (kg/m^2^)	24.9 ± 3.4	24.8 ± 3.4	24.8 ± 3.4	24.9 ± 3.4	25.1 ± 3.3	25.2 ± 3.3	25.3 ± 3.3	25.4 ± 3.3	< 0.001
BMI groups									< 0.001
< 18.5	7456 (2.1%)	6480 (2.1%)	7557 (1.9%)	6856 (1.6%)	5188 (1.3%)	3519 (1.1%)	2004 (0.9%)	2538 (0.9%)	
< 23	89,058 (25.9%)	82,116 (26.8%)	103,622 (26.6%)	107,561 (25.8%)	93,542 (24.6%)	69,772 (23.4%)	45,379 (22.2%)	57,156 (21.4%)	
< 25	83,318 (24.2%)	75,096 (24.5%)	96,421 (24.7%)	103,432 (24.8%)	95,642 (25.1%)	74,654 (25.0%)	50,990 (25.0%)	65,891 (24.7%)	
< 30	139,334 (40.5%)	120,471 (39.3%)	153,721 (39.4%)	167,244 (40.2%)	155,697 (40.9%)	125,535 (42.1%)	87,892 (43.1%)	116,545 (43.7%)	
≥ 30	24,615 (7.1%)	21,679 (7.0%)	28,083 (7.2%)	30,855 (7.4%)	29,960 (7.8%)	24,240 (8.1%)	17,371 (8.5%)	24,087 (9.0%)	
Waist circumference (cm)	85.8 ± 8.7	85.2 ± 8.8	85.1 ± 8.7	85.2 ± 8.7	85.4 ± 8.6	85.5 ± 8.5	85.6 ± 8.4	85.6 ± 8.5	< 0.001
Income, lowest Q1	73,235 (21.3%)	63,875 (20.8%)	80,925 (20.7%)	87,028 (20.9%)	79,498 (20.9%)	62,148 (20.8%)	42,551 (20.9%)	57,194 (21.4%)	< 0.001
Smoking									< 0.001
Non-smoker	168,846 (49.1%)	165,626 (54.1%)	213,368 (54.7%)	229,606 (55.2%)	213,254 (56.1%)	170,622 (57.3%)	120,511 (59.1%)	166,465 (62.5%)	
Ex-smoker	70,670 (20.5%)	60,372 (19.7%)	74,524 (19.1%)	79,039 (19.0%)	70,311 (18.5%)	52,712 (17.7%)	34,019 (16.7%)	39,383 (14.7%)	
Current-smoker	104,265 (30.3%)	79,844 (26.1%)	101,512 (26.0%)	107,303 (25.8%)	96,464 (25.3%)	74,386 (24.9%)	49,106 (24.1%)	60,369 (22.6%)	
Drinking									< 0.001
Non-drinker	175,256 (50.9%)	172,941 (56.5%)	219,889 (56.4%)	235,592 (56.6%)	218,788 (57.5%)	175,221 (58.8%)	123,371 (60.5%)	170,436 (64.0%)	
Mild-drinker	118,004 (34.3%)	99,584 (32.5%)	129,764 (33.3%)	139,985 (33.6%)	126,755 (33.3%)	97,033 (32.5%)	64,152 (31.5%)	76,820 (28.8%)	
Heavy-smoker	50,521 (14.7%)	33,317 (10.8%)	39,751 (10.2%)	40,371 (9.7%)	34,486 (9.0%)	25,466 (8.5%)	16,113 (7.9%)	18,961 (7.1%)	
Regular exercise	73,598 (21.4%)	66,558 (21.7%)	83,520 (21.4%)	87,235 (20.9%)	77,830 (20.4%)	59,518 (19.9%)	39,604 (19.4%)	48,162 (18.0%)	< 0.001
Hypertension	222,974 (64.8%)	186,846 (61.0%)	225,132 (57.8%)	231,510 (55.6%)	205,451 (54.0%)	158,397 (53.2%)	107,310 (52.7%)	140,427 (52.7%)	< 0.001
SBP (mmHg)	128.9 ± 15.8	128.4 ± 15.6	128.5 ± 15.6	128.7 ± 15.7	129 ± 15.7	129.3 ± 15.8	129.6 ± 15.9	130.2 ± 16.3	< 0.001
DBP (mmHg)	78.7 ± 10.4	78.2 ± 10.1	78.5 ± 10.1	78.8 ± 10.2	79.1 ± 10.2	79.4 ± 10.2	79.7 ± 10.3	80.1 ± 10.4	< 0.001
Diabetes mellitus duration, ≥ 5 years	132,768 (38.6%)	114,238 (37.3%)	133,056 (34.1%)	131,108 (31.5%)	110,505 (29.0%)	79,651 (26.7%)	49,963 (24.5%)	58,226 (21.8%)	< 0.001
Use of insulin	41,380 (12.0%)	33,397 (10.9%)	37,117 (9.5%)	35,125 (8.4%)	28,896 (7.6%)	20,834 (7.0%)	13,386 (6.5%)	17,806 (6.6%)	< 0.001
Use of OHA, ≥ 3	64,369 (18.7%)	52,905 (17.3%)	60,506 (15.5%)	59,298 (14.2%)	50,296 (13.2%)	36,981 (12.4%)	23,550 (11.5%)	29,402 (11.0%)	< 0.001
Fasting glucose (mg/dL)	142.4 ± 48.7	140.1 ± 45.3	141.8 ± 45.7	143.5 ± 45.5	145.1 ± 45.9	147.0 ± 46.5	148.9 ± 47.6	152.8 ± 51.2	< 0.001
Use of statin	177,777 (51.7%)	128,879 (42.1%)	119,496 (30.6%)	93,357 (22.4%)	72,261 (19.0%)	60,416 (20.2%)	50,766 (24.9%)	98,495 (37.0%)	< 0.001
Total cholesterol (mg/dL)	149.3 ± 35.5	162.3 ± 23.9	176.1 ± 22	190.3 ± 20.4	204.8 ± 19.5	219.9 ± 19.1	235.2 ± 18.8	268.3 ± 33.6	< 0.001
HDL-cholesterol (mg/dL)	51.6 ± 27.5	51.3 ± 21.6	51.5 ± 21.4	51.7 ± 21.6	51.7 ± 21.6	52.1 ± 23.0	52.6 ± 24.2	54.2 ± 30.2	< 0.001
LDL-cholesterol (mg/dL)	52.3 ± 15.6	77.4 ± 4.2	92.2 ± 4.3	106.9 ± 4.3	121.7 ± 4.3	136.6 ± 4.3	151.4 ± 4.2	185.2 ± 46.4	< 0.001
^a^Triglyceride (mg/dL)	174.5 (174.0–174.9)	142.4 (142.1–142.7)	140.1 (139.9–140.4)	139.9 (139.6–140.1)	140.5 (140.3–140.8)	142.5 (142.2–142.7)	144.4 (144.1–144.7)	151.4 (151.1–151.7)	< 0.001

*BMI* body-mass-index, *DBP* diastolic blood pressure, *HDL* high-density lipoprotein, *LDL* low-density lipoprotein, *OHA* oral hypoglycemic agent, *SBP* systolic blood pressure

^a^Expressed as median (interquartile range)

### LDL-cholesterol and SCA

**Fig. 2:**
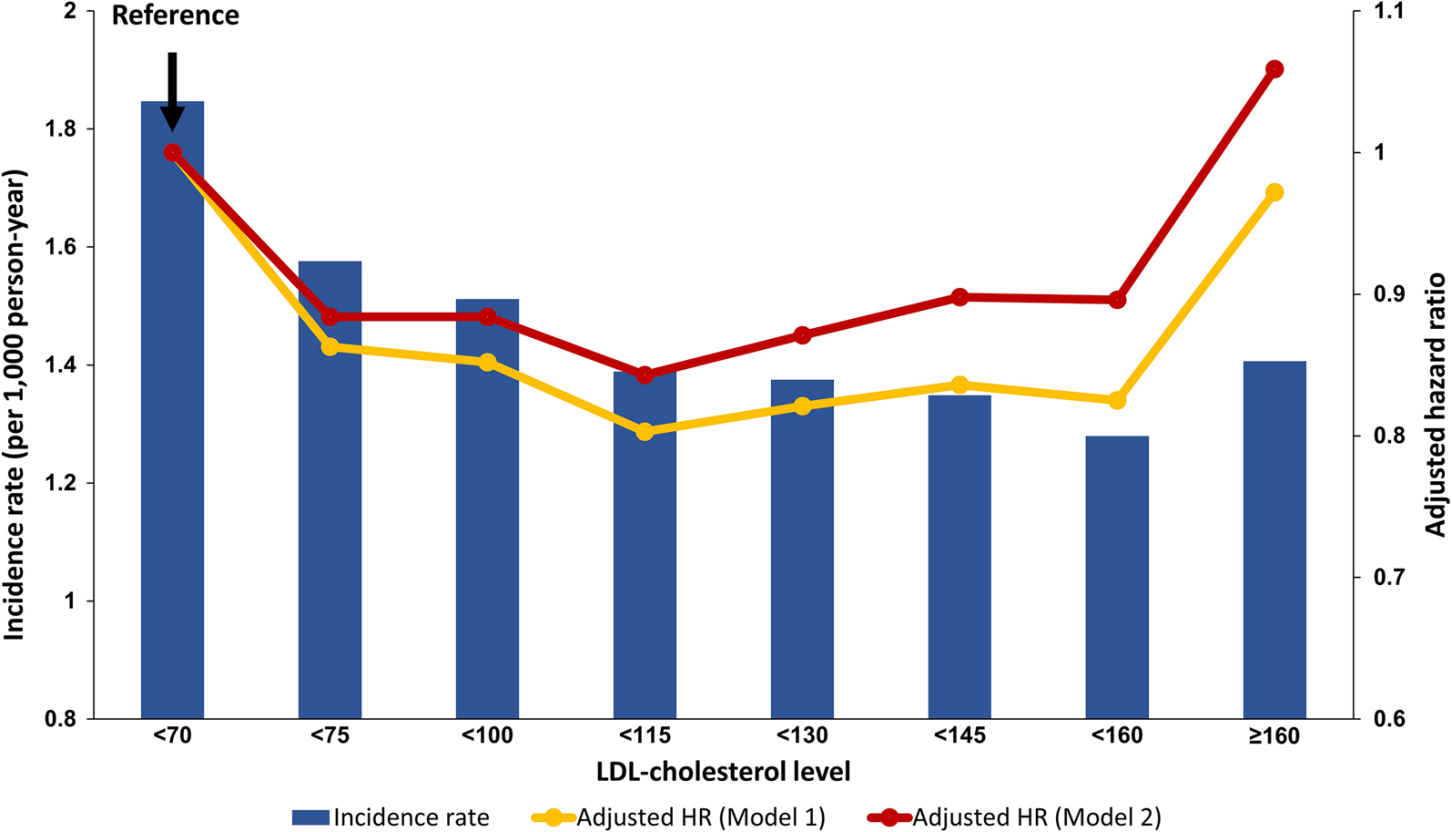
Impact of LDL-cholesterol level on SCA. *HR* hazard ratio, *LDL* low-density lipoprotein, *SCA* sudden cardiac arrest. Hazard ratios were adjusted for age, sex, income, body mass index smoking status, alcohol consumption status, regular exercise, hypertension, fasting blood glucose, duration of diabetes mellitus, use of insulin, use of oral hypoglycemic agent, and use of statin. Incidence is per 1000 person * year follow-up

Before adjustment of covariates, overall incidence of SCA categorized by LDL-cholesterol level was highest in group I (lowest LDL-cholesterol group; incidence = 1.847; Table 2), followed by group II (incidence = 1.575; Table 2), group III (incidence = 1.511; Table 2), and group VIII (highest LDL-cholesterol group; incidence = 1.406; Table 2). After adjustment of age and sex (Model 1), highest risk of SCA was observed in group I, followed by group VIII (Table 2 and Fig. 2). After adjustment of multiple covariates of sociodemographic and metabolic factors (Model 2), risk of SCA was highest in group VIII (adjusted hazard ratio [adjusted-HR] = 1.059, 95% CI = 1.007–1.113; p = 0.020; Table 2 and Fig. 2), followed by group I (reference group; Table 2 and Fig. 2). Before consideration of covariates, overall incidence reflected reverse J-shape relationship with LDL-cholesterol level, whereas adjustment of covariates showed U-shape association, with highest SCA risk in highest LDL-cholesterol group (group VIII). This finding was consistent across age subgroups (Additional file 1: Table S3).

**Table 2:** Impact of LDL-cholesterol level on SCA and subgroup analysis

LDL-cholesterol, mg/dL	N	Event	Duration	IR, per 1000	Adjusted HR
Whole cohort					
< 70	343,781	4313	2,335,003	1.847	1 (Ref.)
< 75	305,842	3283	2,083,277	1.575	0.884 (0.844–0.925)
< 100	389,404	4033	2,668,129	1.511	0.884 (0.846–0.923)
< 115	415,948	3969	2,857,482	1.388	0.843 (0.806–0.881)
< 130	380,029	3602	2,618,974	1.375	0.871 (0.832–0.912)
< 145	297,720	2768	2,052,509	1.348	0.898 (0.855–0.943)
< 160	203,636	1798	1,405,373	1.279	0.896 (0.847–0.947)
≥ 160	266,217	2575	1,831,046	1.406	1.059 (1.007–1.113)
Male					
< 70	235,091	3420	1,587,304	2.154	1 (Ref.)
< 75	192,013	2390	1,299,316	1.839	0.852 (0.809–0.898)
< 100	242,681	2936	1,652,335	1.776	0.861 (0.819–0.905)
< 115	256,338	2838	1,750,195	1.621	0.817 (0.777–0.860)
< 130	227,758	2482	1,560,248	1.590	0.837 (0.794–0.883)
< 145	170,905	1843	1,170,216	1.574	0.867 (0.819–0.919)
< 160	110,355	1152	755,774	1.524	0.880 (0.822–0.941)
≥ 160	127,157	1503	865,297	1.736	1.058 (0.995–1.125)
Female					
< 70	108,690	893	747,698	1.194	1 (Ref.)
< 75	113,829	893	783,961	1.139	1.002 (0.913–1.100)
< 100	146,723	1097	1,015,794	1.079	0.979 (0.895–1.070)
< 115	159,610	1131	1,107,286	1.021	0.943 (0.862–1.032)
< 130	152,271	1120	1,058,726	1.057	0.994 (0.908–1.088)
< 145	126,815	925	882,293	1.048	1.007 (0.916–1.107)
< 160	93,281	646	649,599	0.994	0.973 (0.877–1.079)
≥ 160	139,060	1072	965,748	1.110	1.125 (1.028–1.232)
BMI < 25					
< 70	179,832	2746	1,198,668	2.290	1 (Ref.)
< 75	163,692	2037	1,100,486	1.851	0.843 (0.796–0.893)
< 100	207,600	2486	1,407,077	1.766	0.828 (0.784–0.875)
< 115	217,849	2409	1,482,357	1.625	0.776 (0.734–0.821)
< 130	194,372	2175	1,328,505	1.637	0.804 (0.759–0.852)
< 145	147,945	1604	1,011,792	1.585	0.809 (0.759–0.861)
< 160	98,373	1060	674,577	1.571	0.837 (0.779–0.899)
≥ 160	125,585	1457	857,366	1.699	0.974 (0.913–1.039)
BMI ≥ 25					
< 70	163,949	1567	1,136,334	1.379	1 (Ref.)
< 75	142,150	1246	982,791	1.267	0.952 (0.884–1.026)
< 100	181,804	1547	1,261,051	1.226	0.978 (0.911–1.050)
< 115	198,099	1560	1,375,125	1.134	0.954 (0.888–1.025)
< 130	185,657	1427	1,290,468	1.105	0.979 (0.909–1.054)
< 145	149,775	1164	1,040,716	1.118	1.043 (0.965–1.128)
< 160	105,263	738	730,795	1.009	0.987 (0.902–1.079)
≥ 160	140,632	1118	973,679	1.148	1.194 (1.104–1.292)
Statin naïve					
< 70	166,004	2195	1,124,450	1.952	1 (Ref.)
< 75	176,963	1906	1,200,642	1.587	0.810 (0.761–0.861)
< 100	269,908	2744	1,845,959	1.486	0.781 (0.738–0.826)
< 115	322,591	2959	2,213,922	1.336	0.729 (0.689–0.771)
< 130	307,768	2816	2,117,839	1.329	0.758 (0.717–0.802)
< 145	237,304	2127	1,633,582	1.302	0.784 (0.738–0.832)
< 160	152,870	1316	1,051,772	1.251	0.794 (0.741–0.851)
≥ 160	167,722	1617	1,148,656	1.407	0.963 (0.902–1.028)
Statin use					
< 70	177,777	2118	1,210,552	1.749	1 (Ref.)
< 75	128,879	1377	882,635	1.560	0.954 (0.892–1.021)
< 100	119,496	1289	822,169	1.567	1.020 (0.951–1.093)
< 115	93,357	1010	643,559	1.569	1.062 (0.985–1.145)
< 130	72,261	786	501,135	1.568	1.109 (1.022–1.204)
< 145	60,416	641	418,926	1.530	1.127 (1.031–1.232)
< 160	50,766	482	353,600	1.363	1.060 (0.960–1.171)
≥ 160	98,495	958	682,389	1.403	1.198 (1.108–1.295)
Insulin naïve					
< 70	302,401	3272	2,071,675	1.579	1 (Ref.)
< 75	272,445	2562	1,867,817	1.371	0.886 (0.842–0.934)
< 100	352,287	3204	2,427,033	1.320	0.875 (0.833–0.920)
< 115	380,823	3266	2,628,009	1.242	0.845 (0.804–0.888)
< 130	351,133	2929	2,428,968	1.205	0.849 (0.806–0.893)
< 145	276,886	2299	1,915,834	1.200	0.884 (0.837–0.934)
< 160	190,250	1502	1,316,982	1.140	0.882 (0.829–0.939)
≥ 160	248,411	2123	1,714,647	1.238	1.039 (0.982–1.098)
Insulin use					
< 70	41,380	1041	263,327	3.953	1 (Ref.)
< 75	33,397	721	215,460	3.346	0.874 (0.795–0.962)
< 100	37,117	829	241,096	3.438	0.918 (0.837–1.007)
< 115	35,125	703	229,473	3.063	0.826 (0.749–0.911)
< 130	28,896	673	190,006	3.541	0.978 (0.885–1.080)
< 145	20,834	469	136,675	3.431	0.963 (0.862–1.076)
< 160	13,386	296	88,390	3.348	0.971 (0.852–1.107)
≥ 160	17,806	452	1,16,398	3.883	1.169 (1.045–1.308)

Hazard ratio was adjusted with age, sex, income, body mass index, smoking status, alcohol consumption status, regular exercise, hypertension, fasting blood glucose, duration of diabetes mellitus, use of insulin, use of oral hypoglycemic agent, and use of statin

*LDL* low-density lipoprotein cholesterol, *IR* incidence rate, *HR* hazard ratio, *BMI* body mass index

### Subgroup analysis: gender

**Fig. 3:**
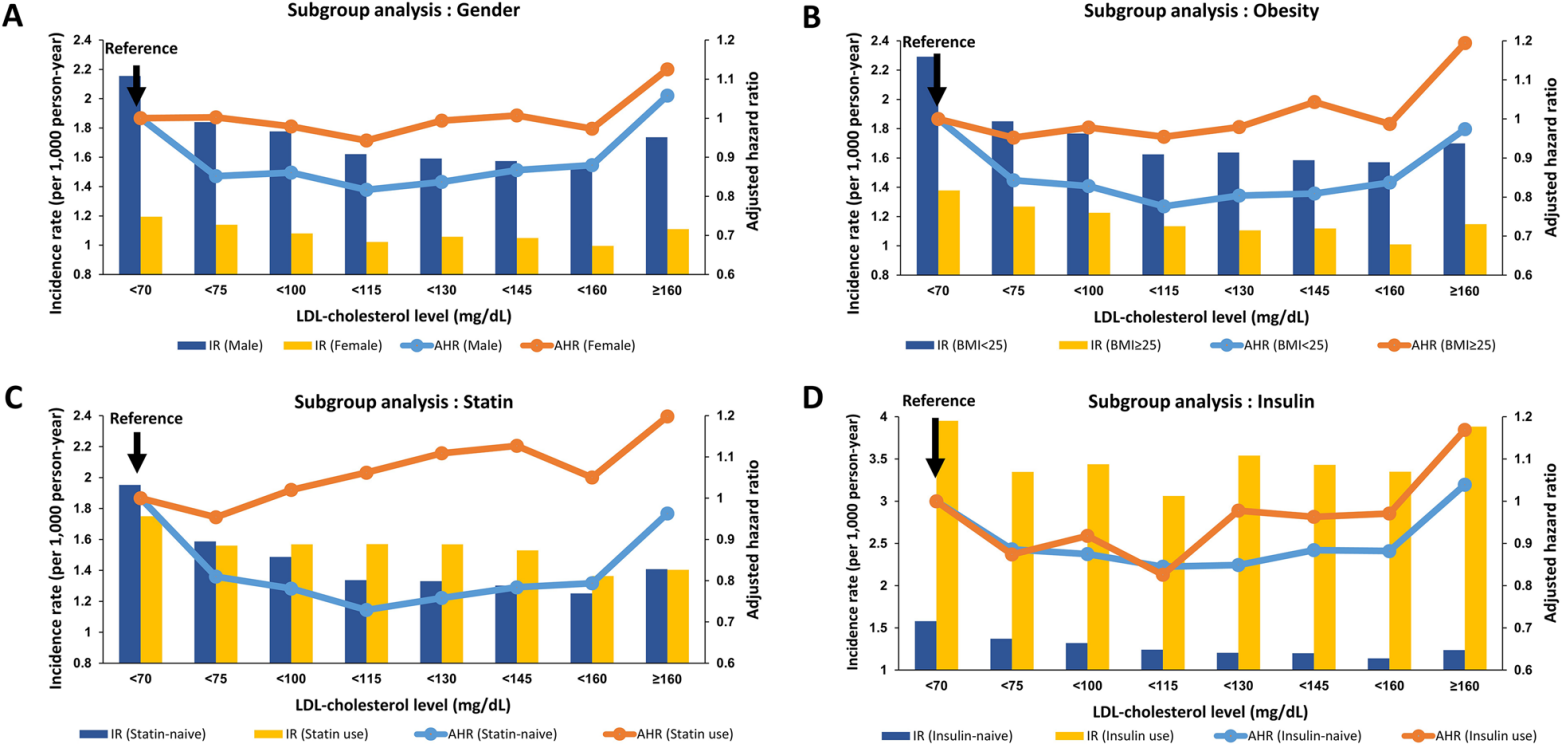
Subgroup analysis according to gender, obesity, use of statin, and use of insulin. *IR* incidence rate, *AHR* adjusted hazard ratio, *LDL* low-density lipoprotein. Hazard ratios were adjusted for age, sex, income, body mass index, smoking status, alcohol consumption status, regular exercise, hypertension, fasting blood glucose, duration of diabetes mellitus, use of insulin, use of oral hypoglycemic agent, and use of statin. Incidence is per 1000 person * year follow-up

The risk of SCA according to LDL-cholesterol level was evaluated in both gender (Table 2 and Fig. 3A). The overall incidence of SCA was significantly higher in male across all LDL-cholesterol subgroups. Both male and female showed similar patterns of SCA incidence, reflecting reverse J-shape curve. After adjustment of covariates, U-shape association was observed in male with highest and lowest LDL-cholesterol group having the higher risk of SCA (Table 2 and Fig. 3A). However, such U-shape association was significantly diluted in female (Table 2 and Fig. 3A).

### Subgroup analysis: obesity

Comparison of obese group (BMI ≥ 25) and non-obese group (BMI < 25) showed markedly lower incidence in obese group regardless of LDL-cholesterol (Table 2 and Fig. 3B). Multivariate model showed U-shape association between LDL-cholesterol level and SCA risk in non-obese group (Table 2 and Fig. 3B). However, such U-shape association was no longer present in the obese group.

### Subgroup analysis: statin

Incidence of SCA regarding the use of statin did not differ significantly (Table 2 and Fig. 3C). In statin-naïve group, adjustment of covariates resulted in prominent U-shape curve, with highest risk of SCA in lowest LDL-cholesterol group (group I; reference group), followed by highest LDL-cholesterol group (group VIII; adjusted-HR = 0.963; 95% CI = 0.902–1.028; Table 2 and Fig. 3C). The risk of SCA in LDL-cholesterol group II to VII was significantly lower than group I. In contrast, the association between LDL-cholesterol and SCA risk was rather linear association in the statin-use group with the highest SCA risk observed in the highest LDL-cholesterol group (adjusted-HR = 1.198; 95% CI = 1.108–1.295; Table 2 and Fig. 3C).

### Subgroup analysis: insulin

Incidence of SCA was significantly higher in diabetes people using insulin compared to insulin-naïve diabetes people, which was consistent across all LDL-cholesterol level (Table 2 and Fig. 3D). In the insulin-naïve group, adjusted-HR was significantly lower in LDL-cholesterol group II to VII and numerically higher in group VIII as compared with the reference group (group I). In the insulin-use group, significantly higher risk of SCA was observed in the highest LDL-cholesterol group (adjusted-HR = 1.169; 95% CI = 1.045–1.308; Table 2 and Fig. 3D).

## Discussion

This study investigated the association of dyslipidemia, in which its severity categorized by LDL-cholesterol level, with the risk of SCA in diabetes patients. Incidence of SCA reflected reverse J-shape association with LDL-cholesterol level: SCA was most prevalent in the lowest LDL-cholesterol group (< 70 mg/dL), and decreased as LDL-cholesterol level rises, until LDL-cholesterol level reached 160 mg/dL. After adjustment of various confounding factors, lowest LDL-cholesterol group (< 70 mg/dL) resulted second highest risk of SCA, following the highest LDL-cholesterol group (≥ 160 mg/dL) resulting in a U-shape association which was contrary finding from traditional linear association of LDL-cholesterol with cardiovascular disease.

This study shows strength in investigating yet undiscovered a U-shape association of SCA and LDL-cholesterol level in diabetes population. Analysis was based on nationwide health insurance database that include large volume of diabetes population, and various patient-related factors were adjusted from vast store of database on sociodemographic information as well as laboratory markers and medication use.

### Sudden cardiac arrest, cardiovascular disease, and dyslipidemia

Sudden cardiac arrest features an abrupt, unexpected onset of cardiac arrest, which may lead to irreversible sequalae even after prompt, successful resuscitation. Primary prevention of SCA with identification and stratification of risk factors have been of concern for decades, but is an area of challenge, due to dynamic course of SCA entangled with various risk factors that influence each other. Dyslipidemia measured with serum LDL-cholesterol level has been identified as a major risk factor for cardiovascular events. Traditionally, LDL-cholesterol level has reflected a positive linear correlation with cardiovascular events [23, 24]. In recent study, lowering of LDL-cholesterol level as low as 40 mg/dL has resulted additional reduction of major cardiovascular events emphasizing reduction of LDL-cholesterol level as low as possible [9, 24]. Moreover, prolonged lowering of LDL-cholesterol is associated with lower risk of atherosclerotic cardiovascular disease [25]. Accordingly, “the lower, the better” paradigm has introduced potent lipid lowering strategies in clinical field, including ezetimibe and proprotein convertase Subtilisin/Kexin 9 inhibitors in addition to statin therapy [26].

### Previous studies on association of LDL-cholesterol and sudden cardiac arrest

In this study, the traditional effect of LDL-cholesterol on cardiovascular mortality was reversed in terms of SCA risk: lowest LDL-cholesterol group showed significantly increased risk of SCA. This reverse association of low LDL-cholesterol level with SCA was even more emphasized in statin-naïve subgroup and non-obese subgroup. Several studies have investigated associations of LDL-cholesterol with the risk of SCA, in which most of them did not find any significant association between LDL-cholesterol and SCA [12, 15]. Hosadurg et al. have reported similar findings with our study: compared with control cohort, out-of-hospital sudden unexpected death cases in North Carolina had significantly lower level of mean total cholesterol, non-high-density lipoprotein cholesterol, and notably, LDL-cholesterol [13]. It had introduced a novel finding of reverse association of low LDL-cholesterol with risk of SCA, but had limitation of small sized samples (n = 399) from geographically limited area, with relatively high proportion of missing values (more than 30%). Our study has further focused on diabetes population, that are more susceptible to dyslipidemia and cardiovascular disease, and confirmed the concept based on large cohort. Prospective nature of the cohort with demonstration of chronological association between LDL-cholesterol level and SCA risk is another strong point of this study.

### Possible mechanisms for reverse association

Although diabetes mellitus and dyslipidemia are known to be independent risk factors for cardiovascular event, it is a cluster of plasma lipid and lipoprotein abnormalities that are metabolically interrelated, known as diabetic dyslipidemia [8]. Therefore, more strict control of lipid level is recommended for prevention of further cardiovascular event in diabetes population. Nevertheless, our study has suggested that low LDL-cholesterol can be associated with paradoxically increased risk of SCA.

The exact mechanisms of the reversal of SCA risk in low LDL-cholesterol group are not established, but it might be supported by several explanations. First, subpopulation of low LDL-cholesterol level (< 70 mg/dL) might represent high risk group for SCA. Patients with pre-existing severe systemic condition (i.e., malnutrition, respiratory disease, inflammatory disease, or malignancy) that is more susceptible to SCA may exhibit low LDL-cholesterol level as a secondary consequence [27]. In contrast to non-obese people, the risk of SCA was not increased in obese people with low LDL-cholesterol suggesting that low LDL-cholesterol can be a surrogate marker of malnutrition. Loss of association between low LDL-cholesterol level and increased risk of SCA in people taking statins also support the hypothesis that low LDL-cholesterol is a surrogate marker for SCA and not a direct determinant. Second possible mechanism is the protective effect of high serum cholesterol on immune system. Several studies have suggested that serum cholesterol plays protective role on bacterial and viral infection by various mechanisms, such as binding to endotoxin, and increase of lymphocytes [28–32]. This protective effect of cholesterol may be more pronounced on diabetes mellitus population, since they are more vulnerable to systemic infection that might cause major organ dysfunction as well as death. The immunomodulatory function of cholesterol also affect the development of virus-related cancer, which may also be related with death [33]. However, this cannot fully explain the phenomenon, since SCA event was confined to out-of-hospital-cardiac-arrest claimed at emergency room, which excludes majority of cancer related death and pre-existing infection. Lastly, undiscovered genetic susceptibility that causes both low LDL-cholesterol and SCA might lie in diabetes patients which needs to be further investigated.

### Clinical implication

The reversed association of SCA risk in low LDL-cholesterol should be carefully interpreted and applied in clinical setting. Although our study revealed the paradoxical increase of SCA risk in low LDL-cholesterol group, this do not imply that low LDL-cholesterol directly cause SCA. On the other hand, prolonged exposure to excessive LDL-cholesterol deteriorates atherosclerotic plaque burden, which provokes significant atherosclerotic cardiovascular disease and associated adverse events. In this regard, result of this study should not change the current therapeutic measurements including statin use and other potent lipid-lowering strategies. However, clinicians should be acknowledged about the increased risk of SCA in low LDL-cholesterol group. They should reconsider whether the low LDL- cholesterol is a surrogate marker of other systemic illness, such as malnutrition or inflammatory disease. In addition, cholesterol plays a key role in maintaining cell membranes and is a precursor for vital substances such as steroid hormone, bile acids, and vitamin D [34]. Although LDL-cholesterol usually refers to deleterious cholesterol that facilitates atherosclerosis, excessively decreased LDL-cholesterol level might indicate decreased protective effect provided by cholesterol. Hence, low LDL-cholesterol itself should not change therapeutic strategies in terms of cardiovascular disease prevention. Nevertheless, potentially increased risk of SCA in diabetic people with low LDL-cholesterol level should be recognized, and corresponding patients can be further stratified of their SCA risk, with comprehending individual’s comorbidities and influence factors.

## Limitations

There are several limitations in this study. First, although SCA risk was adjusted with multiple covariates in our multivariate model which was validated in our previous studies, there can be residual confounders. Second, although the severity of diabetes mellitus was adjusted through prescription of oral hypoglycemic agents or insulin and fasting blood glucose, HbA1c level was not applied for multivariate adjustment. Further encompassment of multifactorial conditions related to diabetes mellitus and dyslipidemia might provide more comprehensive understanding of this reversal of SCA risk in low LDL-cholesterol group. Third, participants included in our study might not represent generalized diabetes population. This study is limited to East Asian population, exclusively confined to South Korean citizen. Since LDL-cholesterol level vary significantly depending on ethnic group, the association between LDL-cholesterol and SCA risk might differ in other populations. Fourth, temporal change in LDL-cholesterol level was not evaluated in this study. Risk of cardiovascular event is influenced not only by the baseline level of LDL-cholesterol but also temporal change of LDL-cholesterol [9, 35]. Association between temporal change in LCL-C and risk of SCA will be an area of future research.

## Conclusions

In people with diabetes mellitus, not only high LDL-cholesterol but also low LDL-cholesterol was associated with increased risk of SCA. Although cause and effect relationship cannot be established based on this study, low LDL-cholesterol can be a surrogate marker for high risk group for SCA among diabetes people. In order to apply this finding to clinical field and preventive measures for SCA, mechanisms for this reversed relationship of LDL-cholesterol and SCA needs to be further investigated.

## Supplementary Information

**Additional file 1: Table S1.** ICD-10 codes for diagnosis. **Table S2.** Definitions of levels of smoking, diabetes mellitus, hypertension, and dyslipidemia used in this study. **Table S3.** Impact of LDL-cholesterol level on sudden cardiac arrest and age-divided subgroup analysis.

## Data Availability

The data underlying this article are available in the article. The raw data underlying this article cannot be shared publicly due to privacy reasons and legal regulations of Republic of Korea. The raw data is stored and analyzed only in the designated server managed by the K-NHIS.

## Abbreviations

Adjusted-HR: Adjusted hazard ratio BMI body mass index
CI: Confidence interval
ICD-10: International Classification of Disease, 10th revision LDL low-density lipoprotein
K-NHIS: Korean National Health Insurance Service
SCA: Sudden cardiac arrest

## References

[1] Al-Khatib SM, Stevenson WG, Ackerman MJ, Bryant WJ, Callans DJ, Curtis AB (2018). 2017 AHA/ACC/HRS guideline for management of patients with ventricular arrhythmias and the prevention of sudden cardiac death: executive summary: a report of the American College of Cardiology/American Heart Association Task Force on clinical practice guidelines and the heart rhythm society. J Am Coll Cardiol.

[2] Priori SG, Blomstrom-Lundqvist C, Mazzanti A, Blom N, Borggrefe M, Camm J (2015). 2015 ESC guidelines for the management of patients with ventricular arrhythmias and the prevention of sudden cardiac death: the task force for the management of patients with ventricular arrhythmias and the prevention of sudden cardiac death of the European society of cardiology (ESC). Endorsed by: Association for European Paediatric and Congenital Cardiology (AEPC). Eur Heart J.

[3] Myerburg RJ, Kessler KM, Castellanos A (1992). Sudden cardiac death. Structure, function, and time-dependence of risk. Circulation.

[4] Norrving B, Puska P, Mendis S, World Health Organization, World Stroke Organization (2011). Global atlas on cardiovascular disease prevention and control.

[5] Chugh SS (2010). Early identification of risk factors for sudden cardiac death. Nat Rev Cardiol.

[6] Kim YG, Roh SY, Han KD, Jeong JH, Choi YY, Min K (2022). Hypertension and diabetes including their earlier stage are associated with increased risk of sudden cardiac arrest. Sci Rep.

[7] Kim YG, Han K, Jeong JH, Roh S-Y, Choi YY, Min K (2022). Metabolic syndrome, gamma-glutamyl transferase, and risk of sudden cardiac death. J Clin Med.

[8] Visseren FLJ, Mach F, Smulders YM, Carballo D, Koskinas KC, Back M (2021). 2021 ESC guidelines on cardiovascular disease prevention in clinical practice. Eur Heart J.

[9] Mach F, Baigent C, Catapano AL, Koskinas KC, Casula M, Badimon L (2020). 2019 ESC/EAS guidelines for the management of dyslipidaemias: lipid modification to reduce cardiovascular risk. Eur Heart J.

[10] Banach M, Surma S, Reiner Z, Katsiki N, Penson PE, Fras Z (2022). Personalized management of dyslipidemias in patients with diabetes—it is time for a new approach (2022). Cardiovasc Diabetol.

[11] Jouven X, Desnos M, Guerot C, Ducimetiere P (1999). Predicting sudden death in the population: the Paris prospective study I. Circulation.

[12] Albert CM, Ma J, Rifai N, Stampfer MJ, Ridker PM (2002). Prospective study of C-reactive protein, homocysteine, and plasma lipid levels as predictors of sudden cardiac death. Circulation.

[13] Hosadurg N, Bogle BM, Joodi G, Sadaf MI, Pursell I, Mendys PM (2018). Lipid profiles in out-of-hospital sudden unexpected death. Mayo Clin Proc Innov Qual Outcomes.

[14] Jouven X, Lemaitre RN, Rea TD, Sotoodehnia N, Empana JP, Siscovick DS (2005). Diabetes, glucose level, and risk of sudden cardiac death. Eur Heart J.

[15] Kurl S, Laaksonen DE, Jae SY, Makikallio TH, Zaccardi F, Kauhanen J (2016). Metabolic syndrome and the risk of sudden cardiac death in middle-aged men. Int J Cardiol.

[16] Kim YG, Han KD, Choi JI, Choi YY, Choi HY, Boo KY (2021). Non-genetic risk factors for atrial fibrillation are equally important in both young and old age: a nationwide population-based study. Eur J Prev Cardiol.

[17] Kim YG, Han KD, Kim DY, Choi YY, Choi HY, Roh SY (2021). Different influence of blood pressure on new-onset atrial fibrillation in pre- and postmenopausal women: a nationwide population-based study. Hypertension.

[18] Kim YG, Han KD, Choi JI, Choi YY, Choi HY, Shim J (2021). Premature ventricular contraction is associated with increased risk of atrial fibrillation: a nationwide population-based study. Sci Rep.

[19] Roh SY, Choi JI, Kim MS, Cho EY, Kim YG, Lee KN (2020). Incidence and etiology of sudden cardiac arrest in Koreans: a cohort from the national health insurance service database. PLoS ONE.

[20] Kim YG, Oh SK, Choi HY, Choi JI (2021). Inherited arrhythmia syndrome predisposing to sudden cardiac death. Korean J Intern Med.

[21] Kim YG, Han KD, Choi JI, Boo KY, Kim DY, Oh SK (2019). The impact of body weight and diabetes on new-onset atrial fibrillation: a nationwide population based study. Cardiovasc Diabetol.

[22] Kim YG, Han KD, Choi JI, Yung Boo K, Kim DY, Oh SK (2019). Impact of the duration and degree of hypertension and body weight on new-onset atrial fibrillation: a nationwide population-based study. Hypertension.

[23] Silverman MG, Ference BA, Im K, Wiviott SD, Giugliano RP, Grundy SM (2016). Association between lowering LDL-C and cardiovascular risk reduction among different therapeutic interventions a systematic review and meta-analysis. JAMA.

[24] Sabatine MS, Giugliano RP, Keech AC, Honarpour N, Wiviott SD, Murphy SA (2017). Evolocumab and clinical outcomes in patients with cardiovascular disease. N Engl J Med.

[25] Ference BA, Ginsberg HN, Graham I, Ray KK, Packard CJ, Bruckert E (2017). Low-density lipoproteins cause atherosclerotic cardiovascular disease. 1. Evidence from genetic, epidemiologic, and clinical studies. A consensus statement from the European atherosclerosis society consensus panel. Eur Heart J.

[26] Wang X, Wen D, Chen Y, Ma L, You C (2022). PCSK9 inhibitors for secondary prevention in patients with cardiovascular diseases: a bayesian network meta-analysis. Cardiovasc Diabetol.

[27] Ravnskov U, Diamond DM, Hama R, Hamazaki T, Hammarskjold B, Hynes N (2016). Lack of an association or an inverse association between low-density-lipoprotein cholesterol and mortality in the elderly: a systematic review. BMJ Open.

[28] Ravnskov U (2003). High cholesterol may protect against infections and atherosclerosis. Qjm Int J Med.

[29] Cavaillon JM, Fitting C, Haeffnercavaillon N, Kirsch SJ, Warren HS (1990). Cytokine response by monocytes and macrophages to free and lipoprotein-bound lipopolysaccharide. Infect Immun.

[30] Weinstock C, Ullrich H, Hohe R, Berg A, Baumstark MW, Frey I (1992). Low-density lipoproteins inhibit endotoxin activation of monocytes. Arterioscler Thromb.

[31] Losche W, Krause S, Pohl A, Pohl C, Liebrenz A, Schauer I (1992). Functional-behavior of mononuclear blood-cells from patients with hypercholesterolemia. Thromb Res.

[32] Muldoon MF, Marsland A, Flory JD, Rabin BS, Whiteside TL, Manuck SB (1997). Immune system differences in men with hypo- or hypercholesterolemia. Clin Immunol Immunopathol.

[33] Read SA, Douglas MW (2014). Virus induced inflammation and cancer development. Cancer Lett.

[34] Zampelas A, Magriplis E (2019). New insights into cholesterol functions: a friend or an enemy?. Nutrients.

[35] Ference BA, Yoo W, Alesh I, Mahajan N, Mirowska KK, Mewada A (2012). Effect of long-term exposure to lower low-density lipoprotein cholesterol beginning early in life on the risk of coronary heart disease a mendelian randomization analysis. J Am Coll Cardiol.

