# Supplementary material for: Association between low-density lipoprotein cholesterol and sudden cardiac arrest in people with diabetes mellitus
Source: Cardiovasc Diabetol. 2023 Feb 20;22:36. doi: 10.1186/s12933-023-01769-9 (PMC9940386; doi:10.1186/s12933-023-01769-9)
Supplement: Supplementary file 1 — Additional file 1: Table S1. ICD-10 codes for diagnosis. Table S2. Definitions of levels of smoking, diabetes mellitus, hypertension, and dyslipidemia used in this study. Table S3. Impact of LDL-cholesterol level on sudden cardiac arrest and age-divided subgroup analysis. [file 12933_2023_1769_MOESM1_ESM.docx]

**Additional file 1**

**Association between Low-density Lipoprotein Cholesterol and Sudden Cardiac Arrest in People with Diabetes Mellitus**

Yun Gi Kim^1*^, Joo Hee Jeong^1*^, Kyung-Do Han^2^, Seung-Young Roh^3^, Kyongjin Min^4^, Hyoung Seok Lee^1^, Yun Young Choi^1^, Jaemin Shim^1^, Jong-Il Choi^1^*, and Young-Hoon Kim^1^

^1^Division of Cardiology, Department of Internal Medicine, Korea University College of Medicine and Korea University Anam Hospital, Seoul, Republic of Korea

^2^Department of Statistics and Actuarial Science, Soongsil University, Seoul, Republic of Korea

^3^Division of Cardiology, Department of Internal Medicine, Korea University College of Medicine and Korea University Guro Hospital, Seoul, Republic of Korea

^4^Division of Cardiology, Sanggye Paik Hospital, Inje University College of Medicine, Seoul, Republic of Korea

**Table S1. ICD-10 codes for diagnosis.**

| **Diseases** | **ICD-10 Codes** |
| --- | --- |
| **Sudden cardiac arrest** | I46.0, I46.1, I46.9, I49.0, R96.0, R96.1 |
| Cardiac arrest with successful resuscitation | I46.0 |
| Sudden cardiac arrest | I46.1 |
| Cardiac arrest, cause unspecified | I46.9 |
| Ventricular fibrillation and flutter | I49.0 |
| Instantaneous death | R96.0 |
| Death occurring less than 24 hours from symptom onset | R96.1 |
| **Hypertension** | I10 – I13, I15 (all sub-codes) |
| **Diabetes mellitus (Type 2)** | E11 – E14 (all sub-codes) |
| **Dyslipidemia** | I78 (all sub-codes) |

ICD-10: International Classification of Diseases, tenth edition.

**Table S2. Definitions of levels of smoking, diabetes mellitus, hypertension, and dyslipidemia used in this study**

|  | Definition |
| --- | --- |
| **Smoking** | |
| Non-smoker | <100 cigarettes in the lifetime |
| Ex-smoker | ≥100 cigarettes in the lifetime, but did not smoke within 1 month of health check-up in 2009 |
| Current smoker | ≥100 cigarettes in the lifetime, and continued smoking within 1 month of health check-up in 2009 |
| **Alcohol consumption** |  |
| Non-drinker | Those who consumed 0 g of alcohol per week |
| Mild-drinker | Those who consumed < 210 g of alcohol per week |
| Heavy-drinker | Those who consumed ≥ 210 g of alcohol per week |
| **Hypertension** | |
| Non-hypertensive | SBP < 120, DBP < 80, and no diagnostic codes for hypertension |
| Hypertension | SBP ≥ 140 or DBP ≥ 90 or diagnostic codes for hypertension |
| **Regular exercise** | Those who had one or more sessions in a week with high (such as running, climbing, intense bicycle activities) or moderate physical activity (such as walking fast, tennis, or moderate bicycle activities). |

DBP: Diastolic blood pressure, mmHg; SBP: Systolic blood pressure, mmHg.

**Table S3. Impact of LDL-cholesterol level on sudden cardiac arrest and age-divided subgroup analysis**

|  | **LDL-cholesterol (mg/dL)** | **IR, per 1000** | **Composite** | | **Subgroup** | |
| --- | --- | --- | --- | --- | --- | --- |
|  |  |  | **Model 1** | **Model 2** | **Model 1** | **Model 2** |
| **Total** | < 70 | 1.847 | 1 (Reference) | 1 (Reference) |  | |
|  | < 75 | 1.575 | 0.863 (0.825 – 0.903) | 0.884 (0.844 – 0.925) |  |  |
|  | < 100 | 1.511 | 0.852 (0.816 – 0.889) | 0.884 (0.846 – 0.923) |  |  |
|  | < 115 | 1.388 | 0.803 (0.770 – 0.839) | 0.843 (0.806 – 0.881) |  |  |
|  | < 130 | 1.375 | 0.821 (0.785 – 0.858) | 0.871 (0.832 – 0.912) |  |  |
|  | < 145 | 1.348 | 0.836 (0.797 – 0.877) | 0.898 (0.855 – 0.943) |  |  |
|  | < 160 | 1.279 | 0.825 (0.781 – 0.872) | 0.896 (0.847 – 0.947) |  |  |
|  | ≥ 160 | 1.406 | 0.972 (0.925 – 1.021) | 1.059 (1.007 – 1.113) |  |  |
| **< 40 years** | < 70 | 0.295 | 1 (Reference) | 1 (Reference) | 1 (Reference) | 1 (Reference) |
|  | < 75 | 0.172 | 0.624 (0.390 – 0.998) | 0.633 (0.396 – 1.012) | 0.624 (0.390 – 0.998) | 0.633 (0.396 – 1.012) |
|  | < 100 | 0.199 | 0.713 (0.473 – 1.074) | 0.742 (0.492 – 1.117) | 0.713 (0.473 – 1.074) | 0.742 (0.492 – 1.117) |
|  | < 115 | 0.239 | 0.835 (0.569 – 1.225) | 0.887 (0.605 – 1.302) | 0.835 (0.569 – 1.225) | 0.887 (0.605 – 1.302) |
|  | < 130 | 0.234 | 0.802 (0.540 – 1.192) | 0.865 (0.582 – 1.285) | 0.802 (0.540 – 1.192) | 0.865 (0.582 – 1.285) |
|  | < 145 | 0.221 | 0.745 (0.483 – 1.150) | 0.807 (0.523 – 1.246) | 0.745 (0.483 – 1.150) | 0.807 (0.523 – 1.246) |
|  | < 160 | 0.180 | 0.603 (0.355 – 1.024) | 0.655 (0.386 – 1.113) | 0.603 (0.355 – 1.024) | 0.655 (0.386 – 1.113) |
|  | ≥ 160 | 0.343 | 1.143 (0.761 – 1.717) | 1.226 (0.816 – 1.842) | 1.143 (0.761 – 1.717) | 1.226 (0.816 – 1.842) |
| **40 - 49 years** | < 70 | 0.880 | 1.419 (1.051 – 1.917) | 1.341 (0.993 – 1.812) | 1 (Reference) | 1 (Reference) |
|  | < 75 | 0.704 | 1.192 (0.875 – 1.624) | 1.174 (0.862 – 1.600) | 0.840 (0.713 – 0.990) | 0.875 (0.743 – 1.031) |
|  | < 100 | 0.530 | 0.910 (0.669 – 1.238) | 0.912 (0.670 – 1.241) | 0.641 (0.545 – 0.754) | 0.680 (0.578 – 0.799) |
|  | < 115 | 0.495 | 0.857 (0.631 – 1.165) | 0.876 (0.644 – 1.191) | 0.604 (0.515 – 0.708) | 0.653 (0.557 – 0.766) |
|  | < 130 | 0.504 | 0.878 (0.646 – 1.195) | 0.914 (0.672 – 1.243) | 0.619 (0.527 – 0.727) | 0.681 (0.580 – 0.801) |
|  | < 145 | 0.505 | 0.887 (0.648 – 1.213) | 0.934 (0.683 – 1.278) | 0.625 (0.526 – 0.742) | 0.697 (0.586 – 0.827) |
|  | < 160 | 0.603 | 1.064 (0.772 – 1.466) | 1.127 (0.818 – 1.552) | 0.750 (0.624 – 0.901) | 0.840 (0.699 – 1.010) |
|  | ≥ 160 | 0.707 | 1.273 (0.935 – 1.732) | 1.331 (0.978 – 1.811) | 0.897 (0.763 – 1.055) | 0.992 (0.844 – 1.167) |
| **50 - 59 years** | < 70 | 1.346 | 1.155 (0.860 – 1.551) | 1.058 (0.788 – 1.421) | 1 (Reference) | 1 (Reference) |
|  | < 75 | 1.003 | 0.895 (0.664 – 1.206) | 0.844 (0.626 – 1.138) | 0.775 (0.699 – 0.859) | 0.798 (0.719 – 0.884) |
|  | < 100 | 0.977 | 0.879 (0.654 – 1.182) | 0.847 (0.630 – 1.140) | 0.761 (0.691 – 0.839) | 0.801 (0.727 – 0.883) |
|  | < 115 | 0.905 | 0.825 (0.613 – 1.109) | 0.814 (0.605 – 1.095) | 0.714 (0.648 – 0.787) | 0.769 (0.698 – 0.848) |
|  | < 130 | 0.782 | 0.726 (0.539 – 0.979) | 0.729 (0.541 – 0.983) | 0.629 (0.567 – 0.697) | 0.689 (0.621 – 0.764) |
|  | < 145 | 0.871 | 0.834 (0.618 – 1.126) | 0.850 (0.630 – 1.147) | 0.722 (0.649 – 0.803) | 0.803 (0.722 – 0.894) |
|  | < 160 | 0.764 | 0.756 (0.556 – 1.028) | 0.783 (0.576 – 1.065) | 0.655 (0.577 – 0.742) | 0.740 (0.652 – 0.840) |
|  | ≥ 160 | 0.852 | 0.907 (0.672 – 1.225) | 0.938 (0.695 – 1.268) | 0.785 (0.704 – 0.876) | 0.887 (0.795 – 0.990) |
| **60 - 69 years** | < 70 | 2.138 | 0.989 (0.732 – 1.336) | 0.913 (0.676 – 1.235) | 1 (Reference) | 1 (Reference) |
|  | < 75 | 1.713 | 0.821 (0.606 – 1.111) | 0.781 (0.576 – 1.057) | 0.830 (0.766 – 0.899) | 0.854 (0.789 – 0.925) |
|  | < 100 | 1.707 | 0.825 (0.610 – 1.115) | 0.796 (0.589 – 1.077) | 0.834 (0.774 – 0.899) | 0.872 (0.808 – 0.940) |
|  | < 115 | 1.603 | 0.779 (0.576 – 1.054) | 0.761 (0.562 – 1.029) | 0.788 (0.730 – 0.850) | 0.833 (0.772 – 0.899) |
|  | < 130 | 1.641 | 0.814 (0.602 – 1.101) | 0.804 (0.594 – 1.088) | 0.823 (0.762 – 0.890) | 0.880 (0.813 – 0.952) |
|  | < 145 | 1.481 | 0.758 (0.559 – 1.028) | 0.760 (0.561 – 1.031) | 0.766 (0.703 – 0.836) | 0.832 (0.763 – 0.909) |
|  | < 160 | 1.494 | 0.797 (0.586 – 1.085) | 0.809 (0.594 – 1.102) | 0.806 (0.730 – 0.890) | 0.886 (0.802 – 0.978) |
|  | ≥ 160 | 1.673 | 0.964 (0.711 – 1.307) | 0.983 (0.725 – 1.334) | 0.975 (0.893 – 1.064) | 1.077 (0.986 – 1.175) |
| **≥ 70 years** | < 70 | 3.763 | 0.944 (0.690 – 1.291) | 0.900 (0.657 – 1.232) | 1 (Reference) | 1 (Reference) |
|  | < 75 | 3.556 | 0.907 (0.663 – 1.242) | 0.879 (0.641 – 1.203) | 0.961 (0.894 – 1.033) | 0.976 (0.908 – 1.050) |
|  | < 100 | 3.596 | 0.918 (0.671 – 1.255) | 0.897 (0.655 – 1.228) | 0.972 (0.908 – 1.041) | 0.997 (0.930 – 1.068) |
|  | < 115 | 3.345 | 0.860 (0.629 – 1.177) | 0.845 (0.617 – 1.157) | 0.912 (0.851 – 0.977) | 0.939 (0.876 – 1.007) |
|  | < 130 | 3.502 | 0.922 (0.674 – 1.261) | 0.915 (0.669 – 1.253) | 0.977 (0.911 – 1.048) | 1.017 (0.947 – 1.092) |
|  | < 145 | 3.521 | 0.956 (0.697 – 1.309) | 0.955 (0.697 – 1.309) | 1.013 (0.939 – 1.092) | 1.061 (0.983 – 1.145) |
|  | < 160 | 3.216 | 0.909 (0.661 – 1.249) | 0.915 (0.666 – 1.258) | 0.963 (0.882 – 1.051) | 1.017 (0.931 – 1.111) |
|  | ≥ 160 | 3.395 | 1.021 (0.744 – 1.400) | 1.036 (0.755 –1.422) | 1.082 (0.999 – 1.172) | 1.151 (1.062 – 1.248) |

LDL: low-density lipoprotein; IR: incidence rate.

Model 1: adjusted for age and sex.

Model 2: age, sex, income, body mass index, smoking status, alcohol consumption status, regular exercise, hypertension, fasting blood glucose, duration of diabetes mellitus, use of insulin, use of oral hypoglycemic agent, and use of statin.
